# Three-Dimensional Environment Sustains Hematopoietic Stem Cell Differentiation into Platelet-Producing Megakaryocytes

**DOI:** 10.1371/journal.pone.0136652

**Published:** 2015-08-27

**Authors:** Audrey Pietrzyk-Nivau, Sonia Poirault-Chassac, Sophie Gandrille, Sidi-Mohammed Derkaoui, Alexandre Kauskot, Didier Letourneur, Catherine Le Visage, Dominique Baruch

**Affiliations:** 1 INSERM, UMR-S 1140, University Paris Descartes, Sorbonne Paris Cité, Paris, France; 2 AP-HP, Georges Pompidou European Hospital, Department of Hematology, Paris, France; 3 INSERM, UMR-S 1148, University Paris Diderot, Paris; University Paris Nord, Villetaneuse, Sorbonne Paris Cité, France; B.C. Cancer Agency, CANADA

## Abstract

Hematopoietic stem cells (HSC) differentiate into megakaryocytes (MK), whose function is to release platelets. Attempts to improve *in vitro* platelet production have been hampered by the low amplification of MK. Providing HSC with an optimal three-dimensional (3D) architecture may favor MK differentiation by mimicking some crucial functions of the bone marrow structure. To this aim, porous hydrogel scaffolds were used to study MK differentiation from HSC as well as platelet production. Flow cytometry, qPCR and perfusion studies showed that 3D was suitable for longer kinetics of CD34^+^ cell proliferation and for delayed megakaryocytic differentiation far beyond the limited shelf-life observed in liquid culture but also increased production of functional platelets. We provide evidence that these 3D effects were related to 1) persistence of MK progenitors and precursors and 2) prolongation of expression of EKLF and c-myb transcription factors involved in early MK differentiation. In addition, presence of abundant mature MK with increased ploidy and impressive cytoskeleton elongations was in line with expression of NF-E2 transcription factor involved in late MK differentiation. Platelets produced in flow conditions were functional as shown by integrin αIIbβ3 activation following addition of exogenous agonists. This study demonstrates that spatial organization and biological cues synergize to improve MK differentiation and platelet production. Thus, 3D environment constitutes a powerful tool for unraveling the physiological mechanisms of megakaryopoiesis and thrombopoiesis in the bone marrow environment, potentially leading to an improved amplification of MK and platelet production.

## Introduction

Hematopoietic stem cells (HSC) isolated from hematopoietic tissues (bone marrow, peripheral blood and cord blood) are self-renewing, multipotent progenitors of hematopoietic lineages leading to all mature blood cells. Structural factors regulating HSC proliferation remain poorly investigated [1]. Inside a tissue structure, cells are organized within the complex molecular framework of the extracellular matrix (ECM). ECM molecules provide tissues with the appropriate three-dimensional (3D) architecture and influence cell migration, proliferation, and differentiation [2]. In order to improve *in vitro* HSC proliferation, 3D structures have been used as physical support to ECM proteins, increasing cell-cell and cell-substrate interactions [3,4].

In the megakaryocytic lineage, megakaryocyte (MK) progenitors proliferate and differentiate into MK precursors that become polyploid by endomitosis, i.e. DNA replication without cell division [5]. Platelets, which are essential for bleeding arrest, are formed from the enlarged cytoplasm of mature MK [5]. MK differentiation occurs *in vitro* in the presence of thrombopoietin (TPO), an essential cytokine for HSC proliferation and differentiation, acting as a MK proliferation, maturation and differentiation agonist [6].

In the bone marrow, interactions between bone cells, hematopoietic cells and vascular cells regulate hematopoiesis. Chemokine-mediated interaction of hematopoietic progenitors with the bone marrow vascular niche is required for thrombopoiesis, the final step of MK differentiation [7]. This process consists of proplatelet formation and platelet release into the circulation from MK fragments entering sinusoid vessels. Platelet release is not synchronized in liquid culture, resulting in a low platelet yield. Platelet production *in vitro* remains a challenging task, requiring synergy between biochemical and biophysical factors to reach high platelet yields. Thus, in the aim to improve *in vitro* platelet production, several novel approaches are currently developed. Hemodynamic shear forces contribute to proplatelet and platelet formation from mature MK *in vivo* [8] and *in vitro* [9]. We reported that platelet production *in vitro* is accelerated and concentrated in flow conditions [10], a process that has been recently reproduced by two others groups [9,11]. A synergy between environment factors has been shown to be involved in i) MK differentiation within the osteoblastic niche of the bone marrow [12], ii) MK migration to the vascular niche [13], and iii) platelet production from fully mature MK [10]. Indeed, most key events occurring during MK maturation appear to be reproduced *ex vivo* in liquid culture, except for the regulatory activities normally provided by the structural bone marrow microenvironment [14]. Even if MK differentiation studies in 3D environment are available [9,11–13,15,16], information is still lacking on the influence of 3D organization not only on thrombopoiesis but also on megakaryopoiesis. 3D biomaterials formed by assembling polymers or biomolecules such as proteins or natural polysaccharides are extensively used in regenerative medicine. Hydrogels have proved valuable for expanding endothelial progenitor cells and for differentiating embryonic stem cells [17,18]. So far, high yields of platelets have been achieved *ex vivo* from HSC using 3D scaffolds of nonwoven polyester fabric and inverted colloidal crystals/polyacrylamide porous hydrogels, but these platelets seemed activated [16]. Nevertheless, MK differentiation leading to this increased platelet production was not investigated in any of these 3D structures.

In this study, we examined how a porous 3D structure affects HSC proliferation, MK differentiation and platelet production. The results clearly demonstrate the effect of 3D environment not only on CD34^+^ cell proliferation but also on MK differentiation. We found that on one hand, 3D environment delayed MK progenitors (CD34^+^/CD41^-^) differentiation into MK precursors (CD34^-^/CD41^+^) leading to mature MK (CD41^+^/CD42b^+^). On the other hand, 3D environment improved enlargement of these mature MK allowing increased platelet production in flow conditions.

## Materials and Methods

### Three-dimensional hydrogels

3D scaffolds were prepared from polysaccharide-based hydrogels as previously described [19,20]. Hydrogels were composed of a 75:25 ratio of pullulan (MW 200 000; Hayashibara Inc, Okayama, Japan) and dextran (MW 500 000; Pharmacia, Uppsala, Sweden) at a concentration of 30% in water (w/v). The resulting hydrogels were porous and were obtained as dry disks of 5 mm in diameter and 1 mm thickness. They were used as a tool to reproduce the 3D environment required for CD34^+^ cell culture in comparison to liquid cell culture.

To study MK differentiation and platelet production in 3D hydrogels, enzymatic lysis of polysaccharides was used to recover the cell suspensions (3D cells). Pullulanase (from *Bacillus acidopullulyticus*, 44.4 U/mL; Sigma-Aldrich, Saint-Quentin Fallavier, France) and dextranase (from *Chaetomium erraticum*, 5.3 U/mL; Sigma-Aldrich) were added to 3D hydrogels for 30 minutes at 37°C, unless otherwise stated. Following scaffold degradation, cells were recovered by centrifugation for 10 minutes at 290*g* and compared to cells cultured in liquid conditions (liquid-culture cells). Controls consisted of liquid-culture cells that were added to empty 3D hydrogels immediately before enzymatic treatment. In some experiments, enzymatic treatment of 3D hydrogels was followed by a bovine serum albumin (BSA; PAA Laboratories, Vélizy Villacoublay, France) density gradient centrifugation to separate matrix debris from viable cells (S1 Fig) [21].

### CD34^+^ cell culture and differentiation

CD34^+^ cells were isolated from human umbilical cord blood (UCB), peripheral blood or bone marrow by using an immunomagnetic technique (Miltenyi Biotec, Paris, France) [22]. This study was approved by the ethics committee/institutional review board “Assistance Publique-Hôpitaux de Paris, Cord Blood Bank, Saint-Louis Hospital, Paris, France” with women’s written informed consent. In order to control for inter-individual variations, CD34^+^ cell samples were separated in two aliquots of 300 000 cells, one for liquid culture and one for 3D hydrogel culture. For liquid culture, 150 000 cells/cm^2^ (Greiner Bio-One, Les Ulis, France) were added and for 3D hydrogel culture, 3 dry disks were seeded with 100 000 cells each (0.2 cm^2^/disk thus 500 000 cells/cm^2^; S1 Fig). The disks were placed in 1-cm^2^ wells, as their volume increased during gradual incorporation of CD34^+^ cells and rehydration by stepwise addition of medium. In both conditions, CD34^+^ cells were cultured at 37°C with 5% CO_2_ in complete medium: Iscove modified Dulbecco’s medium (IMDM; Gibco-Invitrogen, Saint-Aubin, France) supplemented with 15% BIT 9500 serum substitute (Stem Cells Technologies, Grenoble, France), α-monothioglycerol (Sigma-Aldrich) and liposomes (phosphatidyl-choline, cholesterol and oleic acid; Sigma-Aldrich) [22]. Human recombinant stem cell factor (SCF, 20 ng/mL; Miltenyi Biotec) and the thrombopoietin peptide agonist AF13948 named TPO (50 nM) [10] were added once on day 0 to the culture medium, followed by 20 nM TPO alone without any addition of SCF thereafter. In liquid culture, cells were centrifuged and fresh medium with 20 nM TPO was added on days 7, 9 and 12. In 3D hydrogel culture, the disks were transferred to new wells when fresh medium with 20 nM TPO was added, on days 7, 9, 12, 16, 23 and 30 (S1 Fig). When the pores were fully occupied, some cells accumulated at the bottom of the wells. These cells were discarded when the hydrogels were transferred to new wells for medium renewal (S1 Fig). In this way, 3D cells were clearly distinguished from liquid-culture cells.

Online supplemental data available on the PLOS One website described in detail the materials and methods listed below:

- Flow cytometry used for study of differentiation markers and ploidy
- Confocal immunofluorescence microscopy
- Clonogenic assay
- RNA extraction and real-time qPCR
- Platelet production in flow assay
- Platelet activation and spreading
- Statistical data analysis

## Results

### Kinetics of cell growth in 3D environment

**Fig 1:**
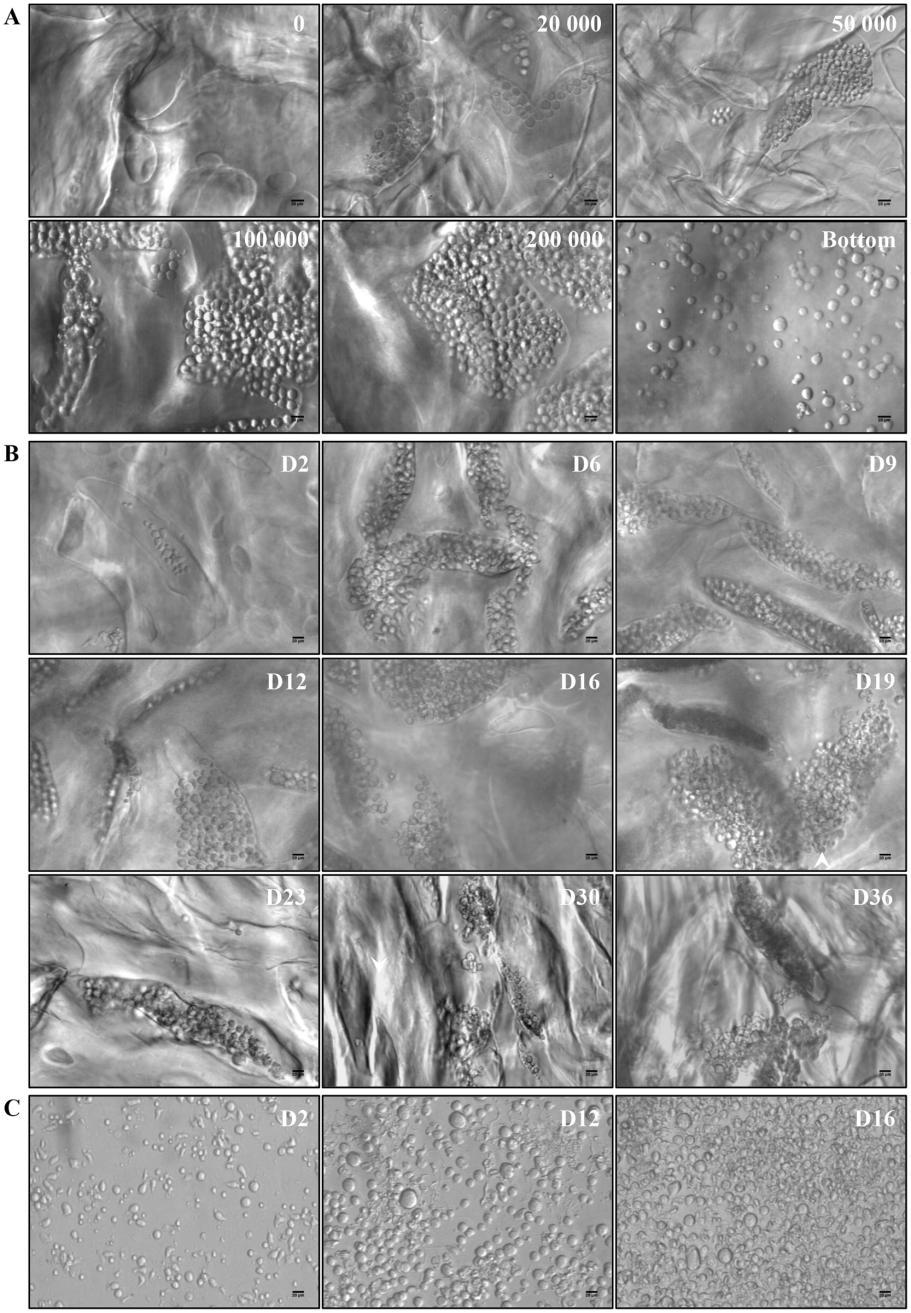
Cell morphology in 3D and in liquid culture. **(A)** Cell proliferation 12 days after cell seeding, relative to the initial cell number/scaffold. **(B)** Cell proliferation and differentiation in 3D between day 2 and day 36. **(C)** Cell proliferation and differentiation in liquid culture on days 2, 12 and 16. All images were acquired using the Axiovert 135 transmission optical microscope with 20X Plasdic magnification. Scale bar = 20 μm.

**Fig 2:**
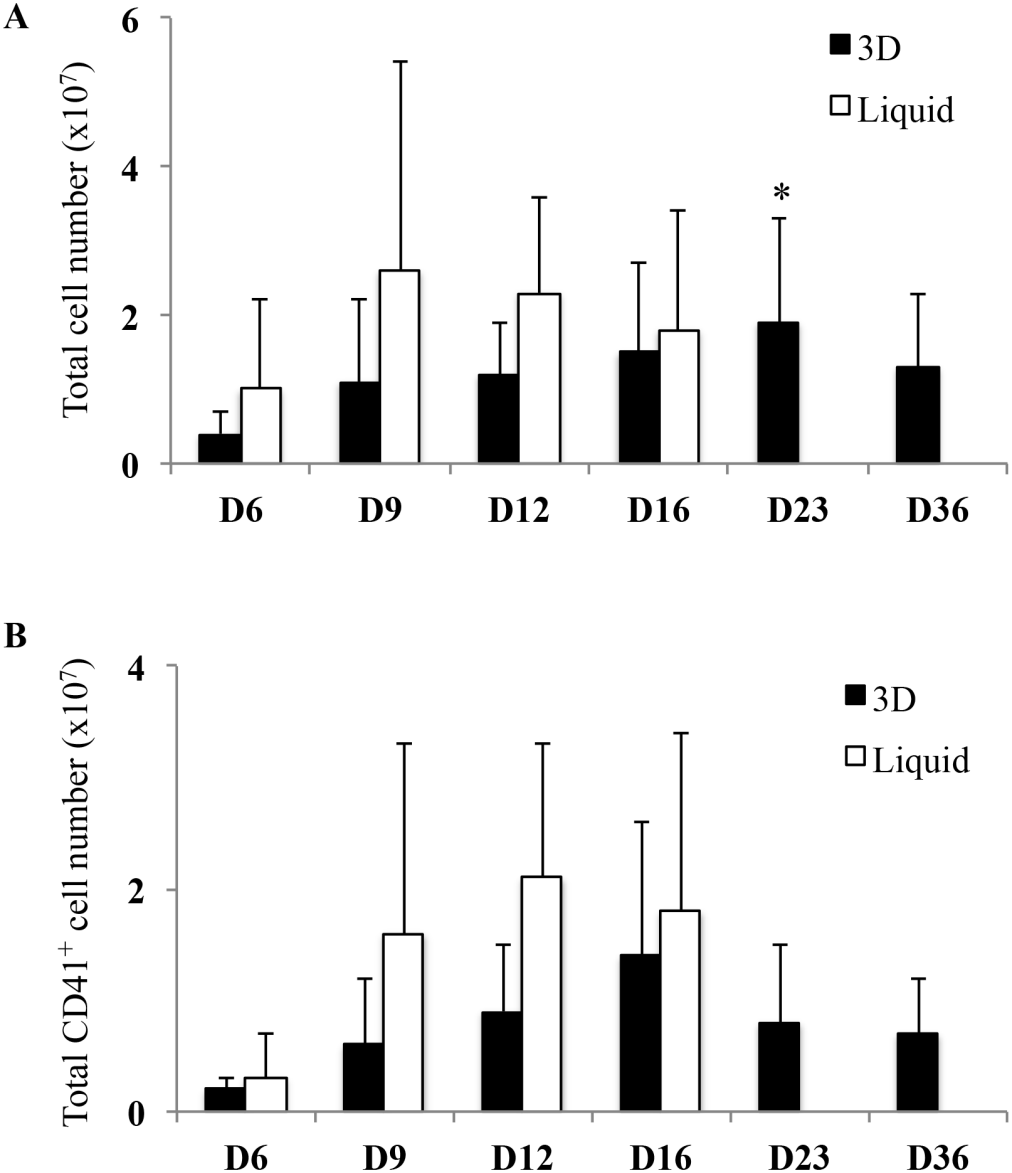
Comparison of total cell number and total CD41^+^ cell number in 3D environment and in liquid culture. **(A)** Histogram representation of total cell number at different days of culture in 3D (black bars) and liquid culture (white bars). Proliferation was determined by cell imaging (Histolab), rather than by colorimetric assay, as polysaccharides produced a high background in the pNPP and MTT assays. Data are means ± SEM of 3 independent experiments. In 3D, late time points (D23 and D36) were compared to day 6. *p<0.05. Some cells exit from hydrogel pores, accumulated at the bottom of the wells (see Fig 1A) and therefore dismissed from the counts. Thus, only cells visible inside the pores of the 3D hydrogel were analyzed. **(B)** Histogram representation of total CD41^+^ cell number in 3D (black bars) and liquid culture (white bars) at different days of culture. Total CD41^+^ cell number was determined by multiplying the total cell number by the frequency of CD41^+^ cells. Data are means ± SEM of 3 independent experiments. In 3D, late time points (D23 and D36) were compared to day 6. Abbreviation: TCN, total cell number.

The 3D environment was designed for optimal CD34^+^ cell proliferation and differentiation. Rehydrated transparent 3D hydrogels were organized into a complex network of large (200 μm) pores (Fig 1A). UCB-derived CD34^+^ cells loaded on top of 3D entered pores within one hour after seeding (not shown). Pore occupancy by proliferating cells was dependent on the initial number of cells, and a condition of 100 000 cells/disk was selected for further experiments (Fig 1A). Cells maintained in 3D survived for at least 36 days (Fig 1B), whereas cells in liquid culture survived for no more than 16 days (Fig 1C). This was confirmed by TUNEL assay performed on day 16 of cells in liquid culture showing many apoptotic cells (data not shown). 3D hydrogel transparency allowed visualizing and studying cell behavior inside pores: cell clusters started to be visible on the second day. Cells proliferated and differentiated, as shown on 9 different microphotographs taken at 3–4 day intervals between the 2^nd^ and 36^th^ day of 3D culture (Fig 1B). In order to estimate kinetics of total cell proliferation, cells grown in 3D and liquid culture were counted by means of image analysis (Fig 2A). Between day 6 and day 23, in 3D, total cell number increased (4.09 x 10^6^ ± 2.29 x 10^6^ on day 6 and 1.89 x 10^7^ ± 1.36 x 10^7^ on day 23; p<0.05) whereas in liquid culture, total cell number increased to reach a peak value of 2.6 x 10^7^ ± 2.75 x 10^7^ on day 9, then decreased until day 16 where total cell number did not differ between the two conditions (1.5 x 10^7^ ± 1.2 x 10^7^ in 3D *vs* 1.8 x 10^7^ ± 1.6 x 10^7^ in liquid culture; NS). After day 16, whereas no cell survived in liquid culture, cells were maintained in 3D up to day 36. Thus, 36 days after initial seeding, large numbers of cells were still present in 3D (Fig 2A). Therefore, an apparent delay in HSC proliferation was observed in 3D that lasted 20 days after the end of cell survival in liquid culture.

To determine whether this delayed cell proliferation resulted in MK differentiation in liquid culture preceding that of cells in 3D, we estimated total CD41^+^ cell (MK precursors) number in both conditions. Results showed a peak of total CD41^+^ cell number on day 12 in liquid culture (2.07 x 10^7^ ± 1.2 x 10^7^) compared with a peak on day 16 in 3D (1.39 x 10^7^ ± 1.2 x 10^7^) followed by MK precursor presence until day 36 (Fig 2B). Taken together, these results indicated that 3D environment was suitable for longer kinetics of CD34^+^ cell proliferation and for delayed megakaryocytic differentiation.

### MK differentiation in 3D environment

**Fig 3:**
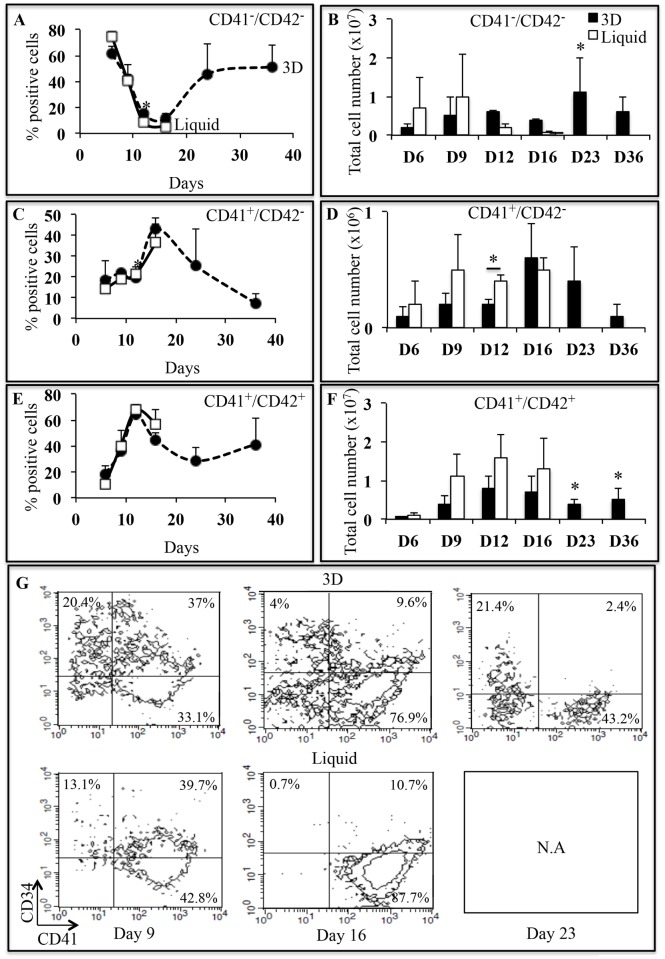
Characterization of MK differentiation as a function of time. **(A, C, E)** Frequency of non-megakaryocytic cells (CD41^-^/CD42b^-^), MK precursors (CD41^+^/CD42b^-^) and mature MK (CD41^+^/CD42b^+^) in 3D (closed circles, dotted lines) and liquid culture (open squares, full lines) between day 6 and day 36. Data are means ± SEM of 3 independent experiments.*p<0.05. In 3D, late time points (D23 and D36) were compared to day 6. **(B)** Histogram representation of total CD41^-^/CD42b^-^ cell number in 3D (black bars) and liquid culture (white bars) at different days of culture. **(D)** Histogram representation of total CD41^+^/CD42b^-^ cell number in 3D (black bars) and liquid culture (white bars) at different days of culture. **(F)** Histogram representation of total CD41^+^/CD42b^+^ cell number in 3D (black bars) and liquid culture (white bars) at different days of culture. Total cell number of each MK population was determined by multiplying the total cell number by the frequency of each MK population. Data are means ± SEM of 3 independent experiments. In 3D, late time points (D23 and D36) were compared to day 7. *p<0.05. **(G)** CD41/CD34 dot plots of one representative experiment of 3 independent experiments in 3D and liquid culture on days 9, 16 and 23. Abbreviation: TCN, total cell number.

To investigate if 3D structure may provide specific information for efficient *in vitro* megakaryopoiesis, UCB-derived CD34^+^ cell differentiation was analyzed by studying the kinetics of CD34, CD41 and CD42b expression (Fig 3). From day 6 to day 15, CD41 and CD42b expression was similar in 3D and liquid culture (Fig 3A–3F), with decreasing percentage of non-megakaryocytic cells (CD41^-^/CD42b^-^; Fig 3A) and increasing percentages of MK precursors (CD41^+^/CD42b^-^; Fig 3C) and of mature MK (CD41^+^/CD42b^+^; Fig 3E). This was also found when total number of non-megakaryocytic cells (Fig 3B), MK precursors (Fig 3D) and mature MK (Fig 3F) were taken into account. A significant population of CD34^+high^ cells with a MFI > 10^3^ was present on day 9 in 3D (11.3%) but absent in liquid culture. Moreover, CD34^+^ cells remained in 3D (31.5% ± 9.7% on day 9 and 6.4% ± 4.9% on day 16) while they disappeared in liquid culture (27.9% ± 12.4%; NS on day 9 and 2.6% ± 2.8%; p<0.05 on day 16) (Fig 3G). Interestingly in 3D from day 16 to day 23, a CD34^+^ cell population persisted (Fig 3G), while CD41 and CD42b were expressed by mature MK (Fig 3E). After day 16, no cells survived in liquid culture as visualized with 7AAD marker (data not shown). These results confirmed the delayed of MK differentiation in 3D environment. Thus, 3D environment provided a suitable environment for maintenance of hematopoietic progenitor CD34^+^ cells.

To confirm more precisely the influence of 3D on non-megakaryocytic cell differentiation, we studied other hematopoietic lineages (S2 Fig). In particular, we identified the presence of myelomonocytic cells (CD11b^+^ and CD11b^+^/CD14^+^ cells) and macrophages (CD14^+^ cells) on days 23 and 36 (Panel A in S2 Fig). In addition, cells expressing the glycophorin A (GpA) erythropoietic marker in 3D persisted during the differentiation between day 6 and day 36 (Panel B in S2 Fig).

**Fig 4:**
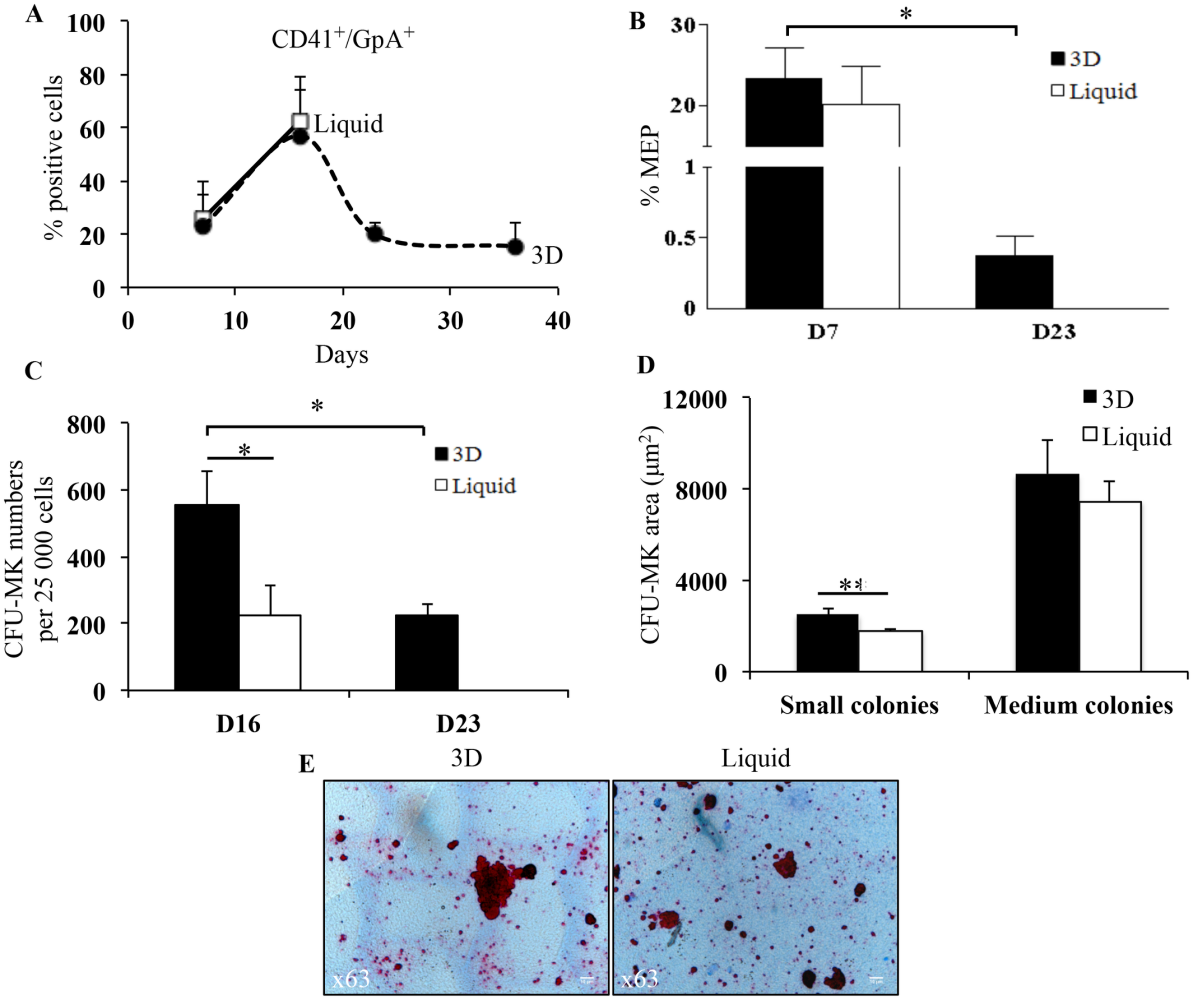
Characterization of CFU-MK and MEP. **(A)** Frequency of CD41^+^/GpA^+^ cells in 3D (closed circles, dotted lines) and liquid culture (open squares, full lines) between day 7 and day 36. Data are means ± SEM of 4 independent experiments. In 3D, late time points (D23 and D36) were compared to day 7. **(B)** Frequency of MEP in 3D (black bars) and liquid culture (white bars) on days 7 and 23. Data are means ± SEM of 4 independent experiments. *p<0.05 between day 7 and day 23 in 3D. **(C)** Total number of CFU-MK produced from the expansion of 25 000 CD34^+^ cells in 3D (black bars) and liquid culture (white bars) on days 16 and 23. Data are means ± SEM of 4 independent experiments.*p<0.05. In 3D, day 23 was compared to day 16. **(D)** Histogram representation of CFU-MK areas on small and medium colonies, in 3D (black bars) and liquid culture (white bars) on day 16. Small and medium colonies were previous classified according to standard classification: small colonies; 3–21 cells/colony and medium colonies; 21–50 cells/colony. Data are means ± SEM of 90 independent colonies. **p<0.01. **(E)** Representative images of CFU-MK areas in 3D and liquid culture on day 16. Images were acquired using the Leica DM40000 B transmission optical microscope with 63X objective. Bar = 10 μm. Abbreviations: CFU-MK, colony forming unit-megakaryocyte; MEP, megakaryocyte-erythroid progenitors.

In addition, in 3D, bipotent progenitors characterized by CD41^+^/GpA^+^ cells [23] persisted at late time points (19.8% ± 4.8% on day 23 and 15.4% ± 8.8% on day 36; Fig 4A). These bipotent progenitors were also identified by studying megakaryocyte-erythroid progenitors (MEP) characterized as CD34^+^/CD41^-^/CD45RA^-^/CD123^-^ cells, 7 and 23 days after seeding. On day 7, the MEP percentage did not differ between the two conditions (23.4% ± 3.8% in 3D and 20.2% ± 4.6% in liquid culture; Fig 4B). On day 23, although decreased compared to day 7, MEP percentage persisted in 3D (0.4% ± 0.1%; p<0.05).

CFU-MK potential was assessed in clonogenic assays performed with 3D cells recovered on days 16 and 23 and with liquid-culture cells recovered on day 16 (Fig 4C and 4D). The number of CFU-MK on day 16 was significantly higher in 3D (556 ± 101) than in liquid culture (224 ± 88; p<0.05) (Fig 4C). On day 23, CFU-MK colonies were still observed in 3D, in numbers similar to those seen in liquid culture on day 16 (Fig 4C). Based on standard classification, no significant difference was observed in the number of small (3–21 cells/colony) and medium (21–50 cells/colony) colonies between both conditions (data not shown). This indicated that cell proliferation was similar in 3D and liquid culture. As shown in Fig 4D and 4E, CFU-MK colonies produced by cells recovered on day 16 were larger in 3D (2497 ± 239 μm^2^ in small colonies and 8645 ± 1522 μm^2^ in medium colonies) than in liquid culture (1765 ± 120 μm^2^; p<0.01 in small colonies and 7446 ± 934 μm^2^; NS in medium colonies). Thus, MK progenitors were able to differentiate into more mature larger MK.

**Fig 5:**
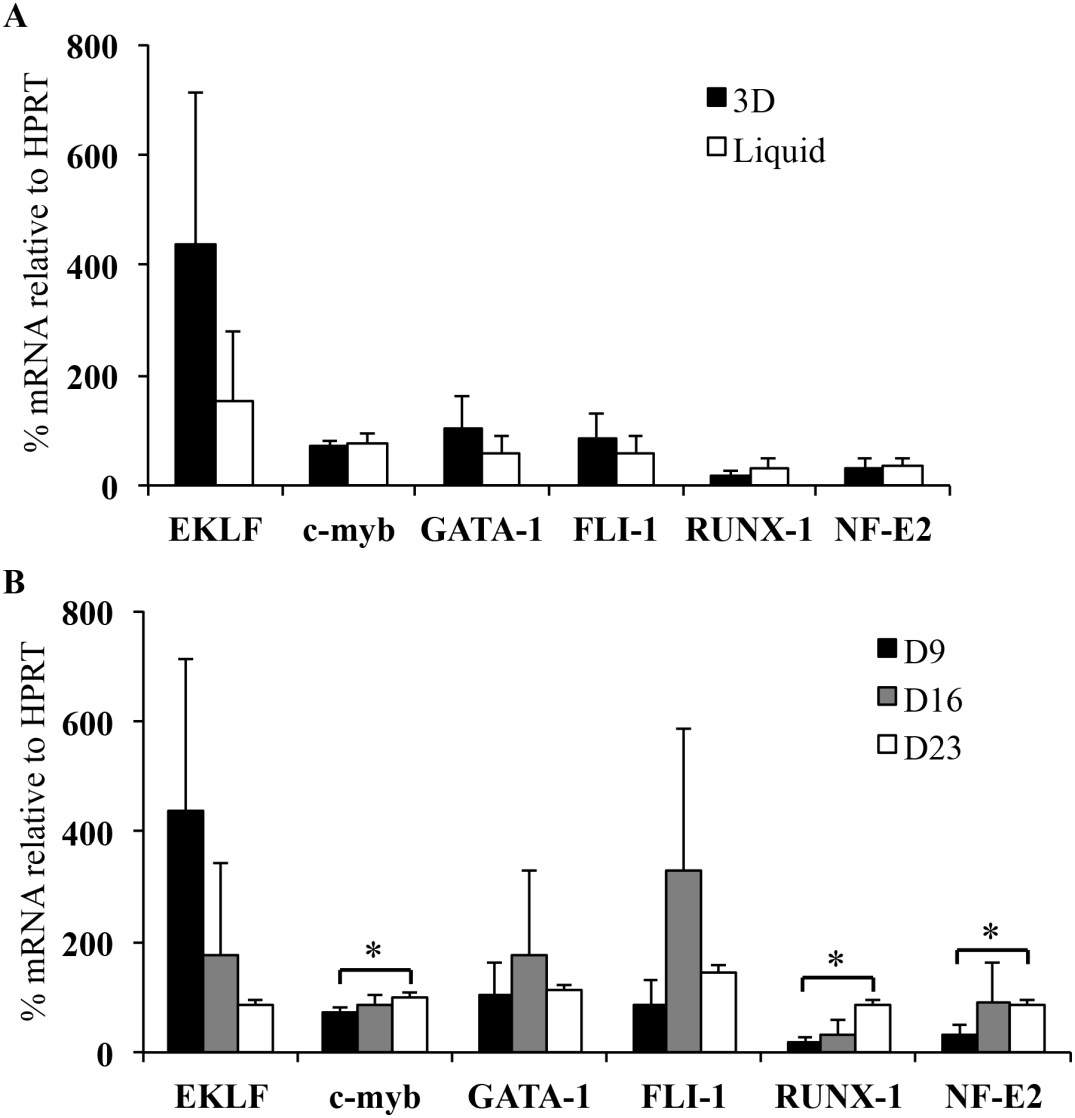
Transcriptional analysis of different genes involved in MK differentiation. **(A)** Histogram representation of the expression levels of various genes determined by RT-qPCR in 3D (black bars) and liquid-culture (white bars) cells at day 9. **(B)** Histogram representation of time-dependent expression levels of various genes determined by RT-qPCR in 3D cells at days 9 (white bars), 16 (gray bars) and 23 (white bars). mRNA percentage of each gene was normalized to that of HPRT mRNA percentage and calibrated via the ΔΔCt method [27]. Data are means ± SEM of 3 independent experiments.*p<0.05. In 3D, days 16 and 23 were compared to day 9. Commercial references of the primer mixes used for qPCR are detailed in S1 Table.

To support our previous results, we analyzed several transcription factors involved in megakaryopoiesis [24–26]. Therefore, we measured by RT-qPCR [27] different gene expression products at day 9 in both conditions and at days 16 and 23 in 3D (Fig 5). On day 9, the expression of EKLF was increased in 3D in comparison to liquid culture. In contrast, the expression of other genes was not different in both conditions (Fig 5A). In 3D, the expression of EKLF involved in early MK differentiation [24,25] decreased between day 9 and day 23 whereas the expression of RUNX-1 and NF-E2 involved in late MK differentiation [24–26] significantly increased (RUNX-1: 17.7% ± 8% on day 9 and 84.9% ± 8.5% on day 23, p<0.05; NF-E2: 31.7% ± 16.2% on day 9 and 87% ± 6.1% on day 23, p<0.05; Fig 5B). c-myb, gene involved in early MK differentiation, also significantly increased between day 9 and day 23 (71.4% ± 9% on day 9 and 97.6% ± 9.4% on day 23; p<0.05). In addition, the expression of GATA-1 and FLI-1 genes, involved in MK progenitor and precursor differentiation [24,25], increased between day 9 and day 16 and then decreased until day 23. The persistence of genes involved in early MK differentiation such as EKLF and c-myb, at late time point (day 23) was in line with the results of flow cytometry (MEP) and clonogenic assay (CFU-MK). Altogether, the persistence of MK progenitors, MK precursors and mature MK associated with late mRNA expression of several transcription factors provided strong evidence that 3D environment support on MK differentiation.

#### Cell ploidy in 3D environment

**Fig 6:**
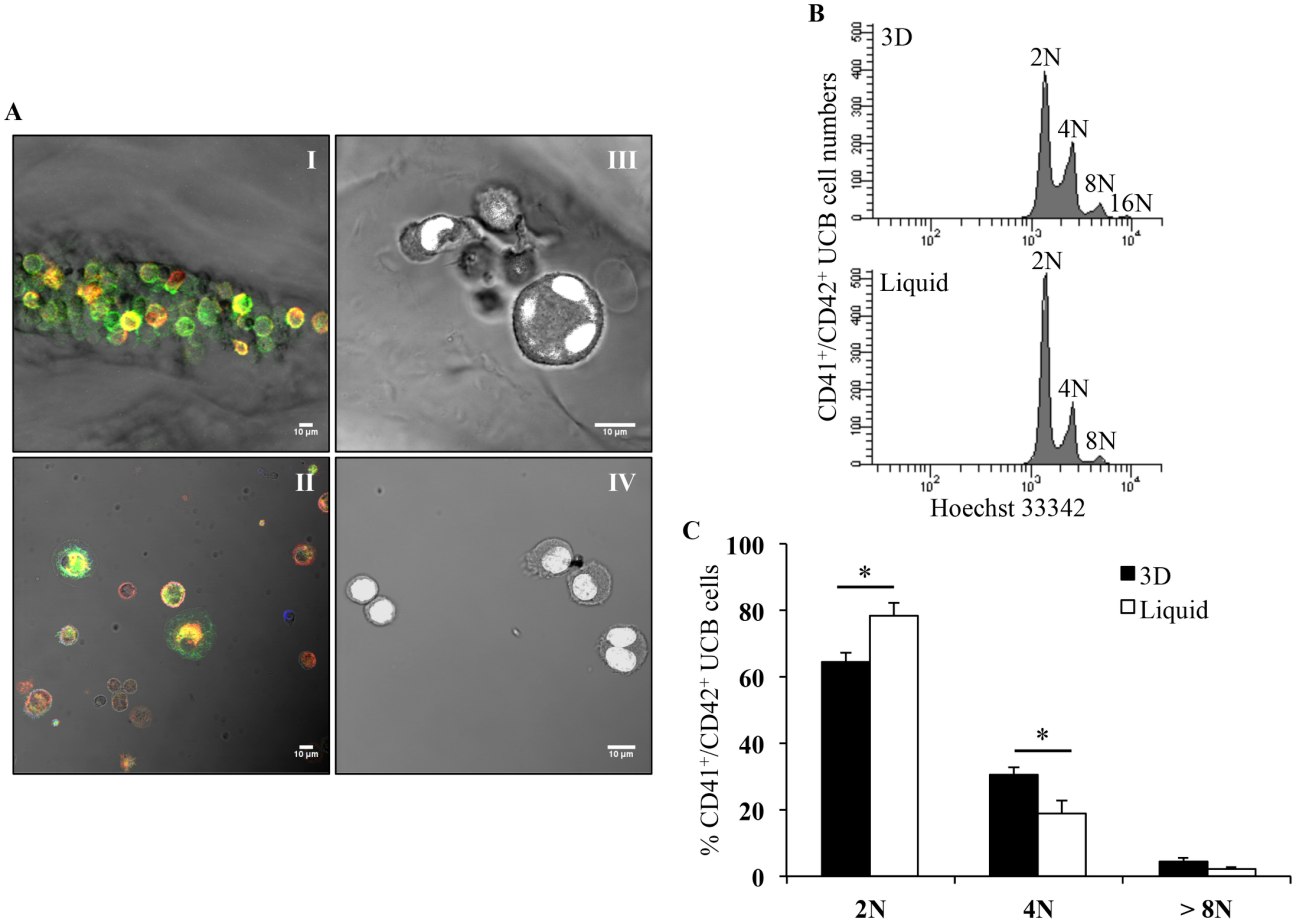
In situ characterization of differentiation markers and ploidy of mature MK. **(A)** Immunofluorescence staining of CD41 (green) and CD42b (red) cells growing inside pores of 3D (I) and in liquid culture (II). Nuclear staining of cell grown in 3D (III) and liquid culture (IV) with YOYO-1 marker (white). All images were acquired 12 days after seeding using the Leica 510 confocal microscope with 40X Plan-NeoFluar objective lens. Scale bar = 10 μm. **(B)** Representative flow cytometry ploidy analysis of CD41^+^/CD42b^+^ UCB cells from 3D and liquid culture, 11 days after seeding. **(C)** Ploidy analysis of CD41^+^/CD42b^+^ UCB cells in 3D (black bars) compared to liquid culture (white bars), 11 days after seeding. Data are means ± SEM of 3 independent experiments. *p<0.05. Abbreviation: UCB, umbilical cord blood.

To better characterize the mechanism of MK differentiation in 3D, UCB CD34^+^ cells were stained within the hydrogels. As shown in Fig 6, 12 days after seeding, MK precursors (CD41^+^/CD42b^-^ cells) and mature MK (CD41^+^/CD42b^+^ cells) were both present in 3D (Fig 6A-I), as in liquid culture (Fig 6A-II). In addition, nuclear staining indicated the presence of tetraploid cells within 3D (Fig 6A-III), contrary to liquid culture where diploid cells were mostly seen (Fig 6A-IV).

Ploidy of UCB mature MK was further investigated 11 days after seeding by flow cytometry assessment of nuclear markers. Cells were recovered from hydrogels by enzymatic degradation of the pullulan-dextran structure in conditions providing the highest recovery while maintaining cell integrity, as determined by microscopy and analysis of surface markers (data not shown). Ploidy levels determined after selection of mature MK (CD41^+^/CD42b^+^ cells selected in singlet cells in division phases) showed a higher ploidy in 3D than in liquid culture (Fig 6B). Cumulative data of three independent experiments showed significantly higher percentages of 4N ploidy classes in 3D mature MK than in liquid-culture mature MK (30.5% ± 2.34% *vs* 19.1% ± 3.45%; p<0.05), whereas 2N ploidy was significantly increased in liquid culture than in 3D (78.1% ± 4.04% *vs* 64.2% ± 2.98%; p<0.05; Fig 6C). The percentage of >8N ploidy classes was not significantly different between both conditions (4.4% ± 0.59% in 3D *vs* 2.3% ± 0.96% in liquid culture; Fig 6C). Moreover, within in each ploidy class, CD41^+^/CD42b^+^ cells were larger when obtained from 3D than from liquid cultures (data not shown). These results showed that the 3D environment enhanced megakaryocytic maturation by favoring endomitosis.

### MK differentiation from adult cells

Culture of adult bone marrow and peripheral blood CD34^+^ cells in 3D environment (S3 Fig) yielded results similar to those obtained with cord blood cells (Panel A in S3 Fig). Twelve days after seeding, the percentages of CD34^+^/CD41^-^ cells (non-megakaryocytic cells and cell progenitors) derived from bone marrow cells (Panel B in S3 Fig, middle) were higher in 3D (25.9%) than in liquid culture (16.9%), as observed with cord blood cells (Panel B in S3 Fig, left). Likewise, using peripheral blood cells, these percentages were also higher in 3D than in liquid culture (32.4% ± *vs* 22.8%; Panel B in S3 Fig, right). Overall, these results showed that 3D environment represents a way to increase the yield of platelet-forming MK derived from both neonatal and adult CD34^+^ cells.

### Mature MK cultured in 3D produce more platelets in flow conditions than liquid-culture MK

**Fig 7:**
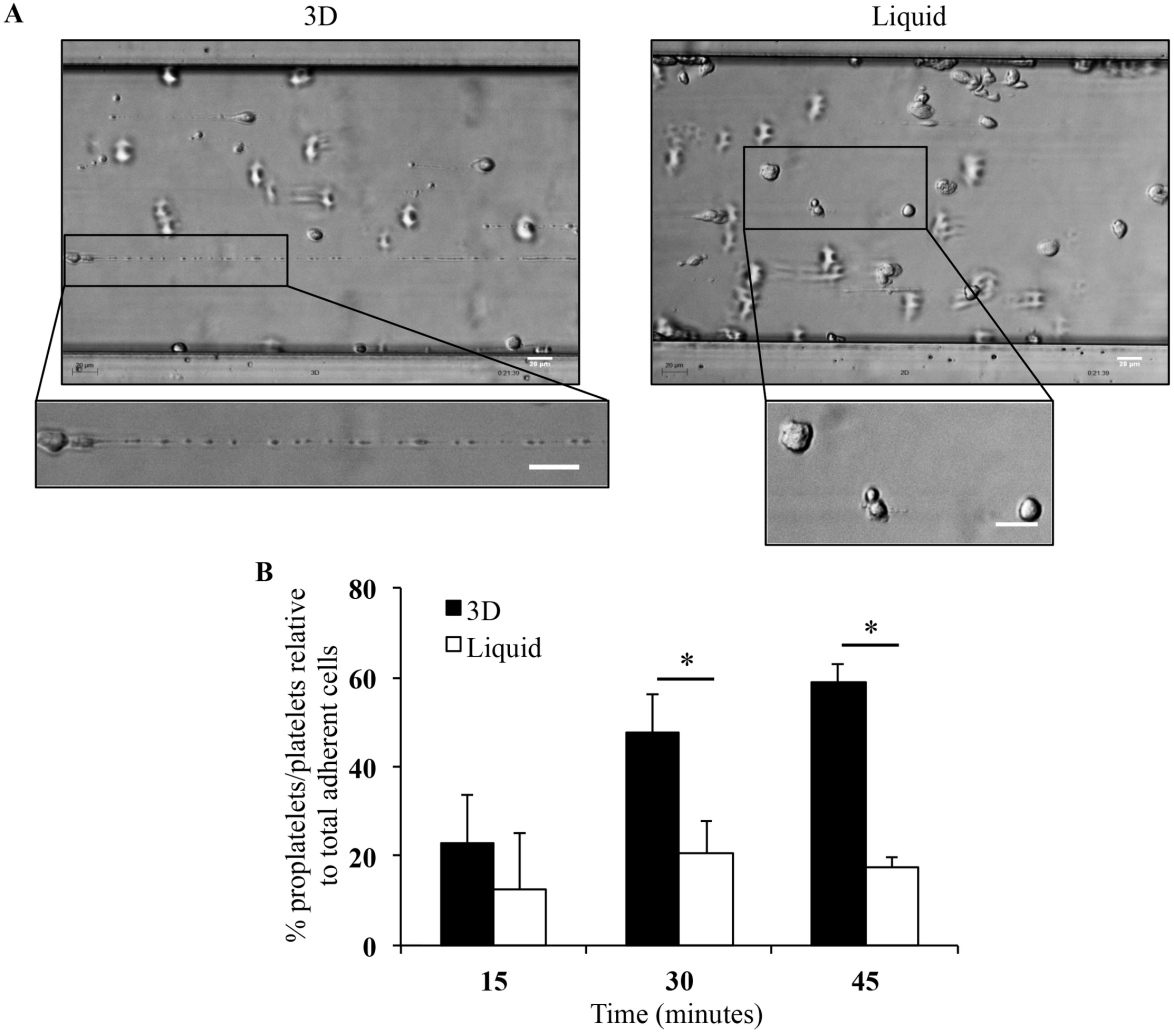
MK deformation and platelet production in flow conditions. **(A)** Stages of MK deformations and reorganization into proplatelets and platelets at 20 minutes of perfusion in 3D (left panel) and in liquid culture (right panel) using Bioflux microfluidic platform to increase platelet formation from mature MK [10]. Images were acquired using the Axiovert 135 transmission optical microscope with 20X Plasdic magnification. Bar = 20 μm. **(B)** Histogram representation of proplatelet/platelet numbers as a percentage of total adherent cells per field recovered from mature MK obtained in 3D (black bars) and liquid culture (white bars). Data are means ± SEM of 3 independent experiments.*p<0.05.

In order to demonstrate that culture in 3D yielded platelets, platelet production was studied in flow conditions after recovery of mature and viable MK from 3D on day 16. When exposed to a high shear rate in a microfluidic platform (S1 Fig), mature MK were able to elongate and to be fragmented into proplatelets and platelets (Fig 7A and S1 Video). As previously shown for platelets derived from liquid-culture MK [10], platelet production from 3D culture MK was upregulated in a flow chamber compared to static conditions (data not shown). Importantly, more and larger cytoskeletal elongations were observed from 3D than liquid-culture mature MK (Fig 7A and S2 Video). This increased ability to elongate resulted in a significantly higher percentage of proplatelets/platelets (59% ± 4.2%) produced by 3D mature MK than liquid-culture MK (17.7% ± 2%, p<0.05; Fig 7B). These data, combined with those in Figs 2A and 3C, indicate that platelet production relative to initial CD34^+^ cell input was higher in 3D (14 platelets/CD34^+^) than in liquid culture (6 platelets/CD34^+^) on day 16. Furthermore, platelet production from 3D mature MK was still observed up to day 36 (data not shown). These results demonstrated that this 3D environment allowed the recovery of many platelets.

## Discussion

HSC isolated from hematopoietic tissues differentiate into MK progenitors and then into mature MK, whose function is to terminally release platelets that are essential for bleeding arrest. To improve *in vitro* platelet production, several attempts are currently developed [9,11,16]. In this study, we demonstrate that the spatial organization conferred by 3D environment sustains an improved MK differentiation, in line with the production of functional platelets.

We demonstrate by structural, functional and transcriptional analyses that the 3D environment provides a delayed of progenitor commitment into MK differentiation, increasing the number of both MK precursors and mature MK, improving the production of functional platelets. Other groups have demonstrated that both CD34^+^ progenitor cell maintenance and expansion were improved by using 3D scaffolds with pore sizes close to 200 μm [3,4,28]. Polysaccharide-based 3D hydrogels with an appropriate composition, structure and elasticity may mimic matrix properties and the bone marrow micro-environment naturally adapted to cell niches [29]. Indeed, porous hydrogels composed of pullulan and dextran displayed several characteristics suitable for studying HSC proliferation and MK differentiation, including their large pore size and high porosity, and also their ability to be enzymatically hydrolyzed [30]. In pullulan/dextran 3D structures, MK progenitors are accommodated inside the large pores favoring cell proximity (Fig 1). This confinement could also concentrate soluble factors such as SCF and TPO favoring CD34^+^ progenitor cell proliferation and MK differentiation. TPO is important not only for HSC and hematopoietic progenitor cell maintenance but also for MK differentiation (reviewed in Kiel and Morrison [31]). In 3D environment, TPO may exert both paracrine and autocrine effects, due to its increased local concentrations [32]. Indeed, cross-linked hydrogels have been shown to bind growth factors and cytokines through ionic interactions with phosphate residues [33]. This confinement was specific to pullulan/dextran 3D hydrogels since we also established that HSC incorporation and MK differentiation were not supported by two other scaffolds: fucoidan cross-linked hydrogels, previously shown to provide proangiogenic activity of endothelial progenitor cells [34], or a fibrin-based matrix shown to enhance HSC proliferation [3] (data not shown). In addition, time-dependent expression of transcription factors in 3D hydrogels confirms longer kinetics of CD34^+^ cell proliferation and the delay of MK differentiation compared with liquid culture. Indeed, the expression of EKLF, c-myb, GATA-1 or FLI-1, involved in early MK differentiation and known to disappear in time in liquid culture [24,25], persisted at late time points in 3D. Furthermore, the expression of transcription factors of early MK progenitors was more important in 3D than in liquid culture. Inversely, the expression of transcription factors of late MK progenitors was more important in liquid culture than in 3D. The second effect of 3D environment is to provide a proliferative advantage of CD34^+^, cells leading to increased numbers of mature MK and to the production of functional platelets. This effect combined with the delayed MK differentiation results in a better maintenance of CD34^+^ cells in 3D environment. This sustained HSC differentiation into MK could be mediated by juxtacrine cellular signaling such as the Notch pathways, by balancing proliferation and differentiation [22,35,36]. Preliminary results based on γ-secretase inhibition by DAPT suggested a Notch pathway implication in the 3D environment (data not shown).

In addition to the specific effect of 3D environment on MK differentiation, our data show that 3D structures support not only MK differentiation but also erythropoietic and myelomonocytic differentiation. Indeed, CD34^+^ cell commitment in other lineages was observed in 3D, as shown by changes of myelomonocytic CD11b and CD14 markers [37,38] and erythropoietic GpA marker [23]; the latter is in line with the persistence of EKLF transcription factor involved in megakaryocyte-erythroid commitment [25]. In addition to endogenously secreted cytokines and growth factors, this effect could be related to SCF persistence within the pores, known to induce CD34^+^ cell commitment in all hematopoietic lineages [39]. Thus, the proliferative advantage of CD34^+^ cells observed in 3D environment is essential to obtain and preserve mature MK but also other mature cells (red blood cells or white blood cells).

The role of polyploidy for increasing platelet formation has been suggested but it still remains unclear [40]. A gene profiling approach established that polyploidization and megakaryocyte differentiation were interrelated in particular *via* increased cytoplasm volume [41]. However so far, platelet formation was not adressed in these studies. Our results indicate that 3D mature MK sustain platelet production until day 36 compared to day 16 in liquid culture. In a step-by-step comparison of MK differentiation in both conditions, our data could establish the influence of MK polyploidization during their maturation, on platelet production. This is based on recent studies showing the link between polyploidization, demarcation membrane system (DMS) and platelet production [42,43]. High levels of ploidy appeared to improve DMS [42] in mature MK, which is at the origin of proplatelet and platelet formation [43]. Interestingly, the high proportion of released platelets in 3D was consistent with several markers related to the maturation of MK. Indeed, we observed larger MK as well as longer elongations of MK cytoskeleton with increased number of proplatelets in flow conditions. In addition, we observed an increased ploidy of UCB- and peripheral blood- mature MK (Panel C in S3 Fig). This shift towards 4N and 8N in 3D suggests that the 3D structure may regulate not only HSC differentiation but also MK ploidy. A link between polyploidization and terminal MK differentiation has been demonstrated involving, for instance, non-muscle myosin IIB heavy chain repression and inhibition of Rho kinase signaling [44,45]. In addition, preliminary results showed that 3D platelets spread over fibrinogen matrix and upon activation with thrombin, a physiological agonist, reorganized their F-actin cytoskeleton (S4 Fig), as show by the formation of filopodia, lamellipodia and stress fiber [46]. This F-actin cytoskeleton reorganization was similar to that of blood platelets, as previously shown [10]. Furthermore, upon activation with the PAR1-activated peptide, the integrin αIIbβ3 modified its conformation and was stained by PAC1 (Panel B in S4 Fig), an antibody binding only to activated platelets [47–50]. Thus, 3D mature MK exposed to high shear rates on a von Willebrand factor are able to produce platelets responding normally to platelet agonists. Most studies of platelet production from MK were focused on MK migration [9,11,13,16]. Recently, alginate/matrigel gels were shown to accommodate mature MK but proplatelet formation was studied in a microfluidic platform *in vitro* without studying the influence of 3D environment on platelet formation [9]. In contrast, our model allows the investigation of 3D environment on both MK differentiation from HSC and platelet production in an *in vitro* microfluidic platform. Thus, this study demonstrates for the first time the effects of 3D environment on MK differentiation and in particular on the improvement of enlargement of mature MK leading to increase the production of platelets. Furthermore, 3D environment is mainly good to keep a continuous supply of CD34^+^ cells to undergo MK differentiation.

Collectively, 3D environment promotes cell clustering, sustains and improves MK differentiation into fully mature MK leading to increase the production of functional platelets. This study provides the first direct evidence that spatial organization and biological cues synergize for efficient MK and platelet production. This 3D environment constitutes a powerful tool for unraveling the physiological mechanisms of megakaryopoiesis and thrombopoiesis in the bone marrow environment. Recent studies showed the feasibility of expanding human induced pluripotent stem cells (iPSCs) into MK underline potential therapeutic applications for generation of platelets [51–53]. Thus, even if a long step forward is necessary between this development and the ultimate goal to provide an alternative to blood-derived platelets, a combination of 3D structures with iPSCs might be of major interest for future therapeutic use.

## Supporting Information

S1 Table Commercial references of the primer mixes used for qPCR. (DOCX)

S1 Fig Schematic outline of steps from 3D cell culture, MK differentiation, mature MK extraction from hydrogels and perfusion, and platelet production. Step 1: CD34^+^ cells were seeded inside 3D pores at day 0 in presence of 50 nM TPO and 20 ng/mL SCF. Steps 2 and 3: On days 7, 9 and 12, the disks were transferred in new wells and fresh medium containing 20 nM TPO was added. Step 4: Cells excluded from 3D and accumulated at the bottom of the wells were discarded. Step 5: On days 16, 23, 30 and 36, mature MK were extracted from 3D by enzymatic lysis. Step 6: Platelets were produced by mature MK perfusion in microchannels coated with VWF. Steps 7 and 8: At the end of the perfusion, platelets were collected and platelet functions were assessed. Abbreviations: TPO, thrombopoietin; SCF, stem cell factor; MK, megakaryocytes; VWF, von Willebrand factor.
(DOCX)

S2 Fig Effect of 3D environment on the expression of myelomonocytic and erythropoietic markers. **(A)** Frequency of CD41^-^/CD11b^+^/CD14^-^, CD41^-^/CD11b^+^/CD14^+^ (both myelomonocytic cells) and CD41^-^/CD11b^-^/CD14^+^ (macrophages) cells in 3D (closed circles, dotted lines) and liquid culture (open squares, full lines) between day 7 and day 36. **(B)** Frequency of CD41^-^/GpA^-^ and CD41^-^/GpA^+^ (erythrocytic cells) cells in 3D (closed circles, dotted lines) and liquid culture (open squares, full lines) between day 7 and day 36. Data are means ± SEM of 4 independent experiments. *p<0.05. In 3D, late time points (days 23 and 36) were compared to day 7. Results indicate that the effect of 3D environment also results in commitment into the erythropoietic and myelomonocytic lineages during a second wave of differentiation that takes place between day 16 and day 36.
(DOCX)

S3 Fig Proliferation and differentiation of neonatal and adult CD34^+^ cells inside 3D environment. **(A)** Bone marrow and peripheral blood cell proliferation inside 3D pores, 12 days after seeding. Images were acquired using the Axiovert 135 transmission optical microscope with 20X Plasdic magnification. Bar = 20 μm. **(B)** CD41/CD34 dot plots of one representative experiment in 3D and liquid culture on day 12. Similar results are obtained with neonatal or adult CD34^+^ cells with the persistence of non-megakaryocytic cells and cell progenitors (CD34^+^/CD41^-^ cells); these cells could still commit in the megakaryocytic lineage. **(C)** Ploidy analysis of CD41^+^/CD42b^+^ peripheral blood cells in 3D (black bars) compared to liquid culture (white bars). Data are means ± SEM of 3 independent experiments. Results show higher percentages of 8N ploidy classes in 3D cells (25.3% ± 6.1%) than in liquid-culture cells (15.9% ± 4.9%), whereas 2N ploidy was more frequent in liquid culture (48.1% ± 6.3%) than in 3D (39.3% ± 6.4%). Abbreviation: UCB, umbilical cord blood.
(DOCX)

S4 Fig Functional properties of 3D platelets collected at the exit of the microchannels. **(A)** Representative images of CD41 (green)/F-actin (red) staining on platelets collected after perfusion of 3D mature MK in microfluidic platform. Filopodia (arrowhead) and stress fibers (arrow) are visible on activated platelets. **(B)** Representative images of PAC1 staining of integrin αIIbβ3 activated (green) and of F-actin staining (red) on platelets collected after perfusion of 3D mature MK in microfluidic platform. Lamellipodia (asterisk) are visible on activated platelets. Images were acquired using the Axio Observer D1 fluorescence optical microscope with 63X Plasdic magnification. Bar = 2 μm.
(DOCX)

S1 Video Platelet production in flow conditions from 3D mature MK. The video shows extremely long MK elongations similar to beads-on-a-thread. Proplatelets and platelets are also visible in the last section of the video. Mature and viable MK were recovered from 3D or liquid culture on day 16 and perfused at a shear rate of 1800 s^-1^ for 45 min on VWF-coated microchannels. Different steps of platelet production were visualized in real-time using the Axiovert 135 transmission optical microscope with 20X Plasdic magnification. Digital images were recorded at 0.25 images/s using Replay software (Microvision Instruments). For visualization, Archimed software (Microvision Instruments) was used to grab frames and record at a velocity of 10 images/sec (40-fold acceleration). Bar = 20 μm.
(DOCX)

S2 Video Platelet production in flow conditions from liquid-culture mature MK. Compared to mature MK obtained from 3D culture, notice the shorter elongations interacting with the surface during the first part of the video and the reduced production of proplatelets and platelets during the second part of the video. Mature and viable MK were recovered from 3D or liquid culture on day 16 and perfused at a shear rate of 1800 s^-1^ for 45 min on VWF-coated microchannels. Different steps of platelet production were visualized in real-time using the Axiovert 135 transmission optical microscope with 20X Plasdic magnification. Digital images were recorded at 0.25 images/s using Replay software (Microvision Instruments). For visualization, Archimed software (Microvision Instruments) was used to grab frames and record at a velocity of 10 images/sec (40-fold acceleration). Bar = 20 μm.
(DOCX)

S1 Materials and Methods (DOCX)
