# Supplementary material for: Three-Dimensional Environment Sustains Hematopoietic Stem Cell Differentiation into Platelet-Producing Megakaryocytes
Source: PLoS One. 2015 Aug 27;10(8):e0136652. doi: 10.1371/journal.pone.0136652 (PMC4552162; doi:10.1371/journal.pone.0136652)
Supplement: S1 Table — (DOCX) [file pone.0136652.s001.docx]

**Three-dimensional environment sustains hematopoietic stem cell differentiation into platelet-producing megakaryocytes**

Audrey Pietrzyk-Nivau^1^, Sonia Poirault-Chassac^1^, Sophie Gandrille^1,2^, Sidi-Mohammed Derkaoui^3^, Alexandre Kauskot^1^, Didier Letourneur^3^, Catherine Le Visage^3^ and Dominique Baruch^1^

^1^INSERM, UMR-S 1140, University Paris Descartes, Sorbonne Paris Cité, Paris, France

^2^AP-HP, Georges Pompidou European Hospital, Department of Hematology, Paris, France

^3^INSERM, UMR-S 1148, University Paris Diderot, Paris; University Paris Nord, Villetaneuse, Sorbonne Paris Cité, France

ONLINE SUPPLEMENTAL DATA

Short title

Increased 3D megakaryocyte and platelet production

Corresponding author

Dr Dominique Baruch

INSERM UMR-S 1140

4 avenue de l’Observatoire, 75006 Paris, France

Mail: dominique.baruch@parisdescartes.fr

Tel: 33 1 53 73 99 38 / Fax: 33 1 44 07 17 72

Supplemental table

S1 Table: Commercial references of the primer mixes used for qPCR

| ***Genes*** | ***Assay ID Human*** |
| --- | --- |
| *EKLF* | Hs00610592_m1 |
| *c-myb* | Hs00920556_m1 |
| *GATA-1* | Hs01085823_m1 |
| *FLI-1* | Hs00956711_m1 |
| *RUNX-1* | Hs00231079_m1 |
| *NF-E2* | Hs0023251_m1 |
| *HPRT* | Hs02800695_m1 |
