# Supplementary material for: Three-Dimensional Environment Sustains Hematopoietic Stem Cell Differentiation into Platelet-Producing Megakaryocytes
Source: PLoS One. 2015 Aug 27;10(8):e0136652. doi: 10.1371/journal.pone.0136652 (PMC4552162; doi:10.1371/journal.pone.0136652)
Supplement: S1 Fig — Step 1: CD34+ cells were seeded inside 3D pores at day 0 in presence of 50 nM TPO and 20 ng/mL SCF. Steps 2 and 3: On days 7, 9 and 12, the disks were transferred in new wells and fresh medium containing 20 nM TPO was added. Step 4: Cells excluded from 3D and accumulated at the bottom of the wells were discarded. Step 5: On days 16, 23, 30 and 36, mature MK were extracted from 3D by enzymatic lysis. Step 6: Platelets were produced by mature MK perfusion in microchannels coated with VWF. Steps 7 and 8: At the end of the perfusion, platelets were collected and platelet functions were assessed. Abbreviations: TPO, thrombopoietin; SCF, stem cell factor; MK, megakaryocytes; VWF, von Willebrand factor. (DOCX) [file pone.0136652.s002.docx]

**Three-dimensional environment sustains hematopoietic stem cell differentiation into platelet-producing megakaryocytes**

Audrey Pietrzyk-Nivau^1^, Sonia Poirault-Chassac^1^, Sophie Gandrille^1,2^, Sidi-Mohammed Derkaoui^3^, Alexandre Kauskot^1^, Didier Letourneur^3^, Catherine Le Visage^3^ and Dominique Baruch^1^

^1^INSERM, UMR-S 1140, University Paris Descartes, Sorbonne Paris Cité, Paris, France

^2^AP-HP, Georges Pompidou European Hospital, Department of Hematology, Paris, France

^3^INSERM, UMR-S 1148, University Paris Diderot, Paris; University Paris Nord, Villetaneuse, Sorbonne Paris Cité, France

ONLINE SUPPLEMENTAL DATA

Short title

Increased 3D megakaryocyte and platelet production

Corresponding author

Dr Dominique Baruch

INSERM UMR-S 1140

4 avenue de l’Observatoire, 75006 Paris, France

Mail: dominique.baruch@parisdescartes.fr

Tel: 33 1 53 73 99 38 / Fax: 33 1 44 07 17 72

Supplemental figures

S1 Fig.: Schematic outline of steps from 3D cell culture, MK differentiation, mature MK extraction from hydrogels and perfusion, and platelet production

Step 1: CD34^+^ cells were seeded inside 3D pores at day 0 in presence of 50 nM TPO and 20 ng/mL SCF.

Steps 2 and 3: On days 7, 9 and 12, the disks were transferred in new wells and fresh medium containing 20 nM TPO was added.

Step 4: Cells excluded from 3D and accumulated at the bottom of the wells were discarded.

Step 5: On days 16, 23, 30 and 36, mature MK were extracted from 3D by enzymatic lysis.

Step 6: Platelets were produced by mature MK perfusion in microchannels coated with VWF.

Steps 7 and 8: At the end of the perfusion, platelets were collected and platelet functions were assessed.

Abbreviations: TPO, thrombopoietin; SCF, stem cell factor; MK, megakaryocytes; VWF, von Willebrand factor.
