# Supplementary material for: Three-Dimensional Environment Sustains Hematopoietic Stem Cell Differentiation into Platelet-Producing Megakaryocytes
Source: PLoS One. 2015 Aug 27;10(8):e0136652. doi: 10.1371/journal.pone.0136652 (PMC4552162; doi:10.1371/journal.pone.0136652)
Supplement: S2 Fig — (A) Frequency of CD41-/CD11b+/CD14-, CD41-/CD11b+/CD14+ (both myelomonocytic cells) and CD41-/CD11b-/CD14+ (macrophages) cells in 3D (closed circles, dotted lines) and liquid culture (open squares, full lines) between day 7 and day 36. (B) Frequency of CD41-/GpA- and CD41-/GpA+ (erythrocytic cells) cells in 3D (closed circles, dotted lines) and liquid culture (open squares, full lines) between day 7 and day 36. Data are means ± SEM of 4 independent experiments. *p<0.05. In 3D, late time points (days 23 and 36) were compared to day 7. Results indicate that the effect of 3D environment also results in commitment into the erythropoietic and myelomonocytic lineages during a second wave of differentiation that takes place between day 16 and day 36. (DOCX) [file pone.0136652.s003.docx]

**Three-dimensional environment sustains hematopoietic stem cell differentiation into platelet-producing megakaryocytes**

Audrey Pietrzyk-Nivau^1^, Sonia Poirault-Chassac^1^, Sophie Gandrille^1,2^, Sidi-Mohammed Derkaoui^3^, Alexandre Kauskot^1^, Didier Letourneur^3^, Catherine Le Visage^3^ and Dominique Baruch^1^

^1^INSERM, UMR-S 1140, University Paris Descartes, Sorbonne Paris Cité, Paris, France

^2^AP-HP, Georges Pompidou European Hospital, Department of Hematology, Paris, France

^3^INSERM, UMR-S 1148, University Paris Diderot, Paris; University Paris Nord, Villetaneuse, Sorbonne Paris Cité, France

ONLINE SUPPLEMENTAL DATA

Short title

Increased 3D megakaryocyte and platelet production

Corresponding author

Dr Dominique Baruch

INSERM UMR-S 1140

4 avenue de l’Observatoire, 75006 Paris, France

Mail: dominique.baruch@parisdescartes.fr

Tel: 33 1 53 73 99 38 / Fax: 33 1 44 07 17 72

Supplemental figures

S2 Fig.: Effect of 3D environment on the expression of myelomonocytic and erythropoietic markers

**(A)** Frequency of CD41^-^/CD11b^+^/CD14^-^, CD41^-^/CD11b^+^/CD14^+^ (both myelomonocytic cells) and CD41^-^/CD11b^-^/CD14^+^ (macrophages) cells in 3D (closed circles, dotted lines) and liquid culture (open squares, full lines) between day 7 and day 36. **(B)** Frequency of CD41^-^/GpA^-^ and CD41^-^/GpA^+^ (erythrocytic cells) cells in 3D (closed circles, dotted lines) and liquid culture (open squares, full lines) between day 7 and day 36. Data are means ± SEM of 4 independent experiments. *p<0.05. In 3D, late time points (days 23 and 36) were compared to day 7. Results indicate that the effect of 3D environment also results in commitment into the erythropoietic and myelomonocytic lineages during a second wave of differentiation that takes place between day 16 and day 36.
