# Supplementary material for: Three-Dimensional Environment Sustains Hematopoietic Stem Cell Differentiation into Platelet-Producing Megakaryocytes
Source: PLoS One. 2015 Aug 27;10(8):e0136652. doi: 10.1371/journal.pone.0136652 (PMC4552162; doi:10.1371/journal.pone.0136652)
Supplement: S3 Fig — (A) Bone marrow and peripheral blood cell proliferation inside 3D pores, 12 days after seeding. Images were acquired using the Axiovert 135 transmission optical microscope with 20X Plasdic magnification. Bar = 20 μm. (B) CD41/CD34 dot plots of one representative experiment in 3D and liquid culture on day 12. Similar results are obtained with neonatal or adult CD34+ cells with the persistence of non-megakaryocytic cells and cell progenitors (CD34+/CD41- cells); these cells could still commit in the megakaryocytic lineage. (C) Ploidy analysis of CD41+/CD42b+ peripheral blood cells in 3D (black bars) compared to liquid culture (white bars). Data are means ± SEM of 3 independent experiments. Results show higher percentages of 8N ploidy classes in 3D cells (25.3% ± 6.1%) than in liquid-culture cells (15.9% ± 4.9%), whereas 2N ploidy was more frequent in liquid culture (48.1% ± 6.3%) than in 3D (39.3% ± 6.4%). Abbreviation: UCB, umbilical cord blood. (DOCX) [file pone.0136652.s004.docx]

**Three-dimensional environment sustains hematopoietic stem cell differentiation into platelet-producing megakaryocytes**

Audrey Pietrzyk-Nivau^1^, Sonia Poirault-Chassac^1^, Sophie Gandrille^1,2^, Sidi-Mohammed Derkaoui^3^, Alexandre Kauskot^1^, Didier Letourneur^3^, Catherine Le Visage^3^ and Dominique Baruch^1^

^1^INSERM, UMR-S 1140, University Paris Descartes, Sorbonne Paris Cité, Paris, France

^2^AP-HP, Georges Pompidou European Hospital, Department of Hematology, Paris, France

^3^INSERM, UMR-S 1148, University Paris Diderot, Paris; University Paris Nord, Villetaneuse, Sorbonne Paris Cité, France

ONLINE SUPPLEMENTAL DATA

Short title

Increased 3D megakaryocyte and platelet production

Corresponding author

Dr Dominique Baruch

INSERM UMR-S 1140

4 avenue de l’Observatoire, 75006 Paris, France

Mail: dominique.baruch@parisdescartes.fr

Tel: 33 1 53 73 99 38 / Fax: 33 1 44 07 17 72

Supplemental figures

S3 Fig.: Proliferation and differentiation of neonatal and adult CD34^+^ cells inside 3D environment

**(A)** Bone marrow and peripheral blood cell proliferation inside 3D pores, 12 days after seeding. Images were acquired using the Axiovert 135 transmission optical microscope with 20X Plasdic magnification. Bar = 20 µm. **(B)** CD41/CD34 dot plots of one representative experiment in 3D and liquid culture on day 12. Similar results are obtained with neonatal or adult CD34^+^ cells with the persistence of non-megakaryocytic cells and cell progenitors (CD34^+^/CD41^-^ cells); these cells could still commit in the megakaryocytic lineage. **(C)** Ploidy analysis of CD41^+^/CD42b^+^ peripheral blood cells in 3D (black bars) compared to liquid culture (white bars). Data are means ± SEM of 3 independent experiments. Results show higher percentages of 8N ploidy classes in 3D cells (25.3% ± 6.1%) than in liquid-culture cells (15.9% ± 4.9%), whereas 2N ploidy was more frequent in liquid culture (48.1% ± 6.3%) than in 3D (39.3% ± 6.4%). Abbreviation: UCB, umbilical cord blood.
