# Supplementary material for: Three-Dimensional Environment Sustains Hematopoietic Stem Cell Differentiation into Platelet-Producing Megakaryocytes
Source: PLoS One. 2015 Aug 27;10(8):e0136652. doi: 10.1371/journal.pone.0136652 (PMC4552162; doi:10.1371/journal.pone.0136652)
Supplement: S4 Fig — (A) Representative images of CD41 (green)/F-actin (red) staining on platelets collected after perfusion of 3D mature MK in microfluidic platform. Filopodia (arrowhead) and stress fibers (arrow) are visible on activated platelets. (B) Representative images of PAC1 staining of integrin αIIbβ3 activated (green) and of F-actin staining (red) on platelets collected after perfusion of 3D mature MK in microfluidic platform. Lamellipodia (asterisk) are visible on activated platelets. Images were acquired using the Axio Observer D1 fluorescence optical microscope with 63X Plasdic magnification. Bar = 2 μm. (DOCX) [file pone.0136652.s005.docx]

**Three-dimensional environment sustains hematopoietic stem cell differentiation into platelet-producing megakaryocytes**

Audrey Pietrzyk-Nivau^1^, Sonia Poirault-Chassac^1^, Sophie Gandrille^1,2^, Sidi-Mohammed Derkaoui^3^, Alexandre Kauskot^1^, Didier Letourneur^3^, Catherine Le Visage^3^ and Dominique Baruch^1^

^1^INSERM, UMR-S 1140, University Paris Descartes, Sorbonne Paris Cité, Paris, France

^2^AP-HP, Georges Pompidou European Hospital, Department of Hematology, Paris, France

^3^INSERM, UMR-S 1148, University Paris Diderot, Paris; University Paris Nord, Villetaneuse, Sorbonne Paris Cité, France

ONLINE SUPPLEMENTAL DATA

Short title

Increased 3D megakaryocyte and platelet production

Corresponding author

Dr Dominique Baruch

INSERM UMR-S 1140

4 avenue de l’Observatoire, 75006 Paris, France

Mail: dominique.baruch@parisdescartes.fr

Tel: 33 1 53 73 99 38 / Fax: 33 1 44 07 17 72

Supplemental figures

S4 Fig.: Functional properties of 3D platelets collected at the exit of the microchannels

**(A)** Representative images of CD41 (green)/F-actin (red) staining on platelets collected after perfusion of 3D mature MK in microfluidic platform. Filopodia (arrowhead) and stress fibers (arrow) are visible on activated platelets. **(B)** Representative images of PAC1 staining of integrin αIIbβ3 activated (green) and of F-actin staining (red) on platelets collected after perfusion of 3D mature MK in microfluidic platform. Lamellipodia (asterisk) are visible on activated platelets. Images were acquired using the Axio Observer D1 fluorescence optical microscope with 63X Plasdic magnification. Bar = 2 µm.
