# Supplementary material for: Three-Dimensional Environment Sustains Hematopoietic Stem Cell Differentiation into Platelet-Producing Megakaryocytes
Source: PLoS One. 2015 Aug 27;10(8):e0136652. doi: 10.1371/journal.pone.0136652 (PMC4552162; doi:10.1371/journal.pone.0136652)
Supplement: S1 Video — The video shows extremely long MK elongations similar to beads-on-a-thread. Proplatelets and platelets are also visible in the last section of the video. Mature and viable MK were recovered from 3D or liquid culture on day 16 and perfused at a shear rate of 1800 s-1 for 45 min on VWF-coated microchannels. Different steps of platelet production were visualized in real-time using the Axiovert 135 transmission optical microscope with 20X Plasdic magnification. Digital images were recorded at 0.25 images/s using Replay software (Microvision Instruments). For visualization, Archimed software (Microvision Instruments) was used to grab frames and record at a velocity of 10 images/sec (40-fold acceleration). Bar = 20 μm. (DOCX) [file pone.0136652.s006.docx]

**Three-dimensional environment sustains hematopoietic stem cell differentiation into platelet-producing megakaryocytes**

Audrey Pietrzyk-Nivau^1^, Sonia Poirault-Chassac^1^, Sophie Gandrille^1,2^, Sidi-Mohammed Derkaoui^3^, Alexandre Kauskot^1^, Didier Letourneur^3^, Catherine Le Visage^3^ and Dominique Baruch^1^

^1^INSERM, UMR-S 1140, University Paris Descartes, Sorbonne Paris Cité, Paris, France

^2^AP-HP, Georges Pompidou European Hospital, Department of Hematology, Paris, France

^3^INSERM, UMR-S 1148, University Paris Diderot, Paris; University Paris Nord, Villetaneuse, Sorbonne Paris Cité, France

ONLINE SUPPLEMENTAL DATA

Short title

Increased 3D megakaryocyte and platelet production

Corresponding author

Dr Dominique Baruch

INSERM UMR-S 1140

4 avenue de l’Observatoire, 75006 Paris, France

Mail: dominique.baruch@parisdescartes.fr

Tel: 33 1 53 73 99 38 / Fax: 33 1 44 07 17 72

Supplemental videos

S1 and S2 Videos

Mature and viable MK were recovered from 3D or liquid culture on day 16 and perfused at a shear rate of 1800 s^-1^ for 45 min on VWF-coated microchannels. Different steps of platelet production were visualized in real-time using the Axiovert 135 transmission optical microscope with 20X Plasdic magnification. Digital images were recorded at 0.25 images/s using Replay software (Microvision Instruments). For visualization, Archimed software (Microvision Instruments) was used to grab frames and record at a velocity of 10 images/sec (40-fold acceleration). Bar = 20 µm.

S1 Video: Platelet production in flow conditions from 3D mature MK

The video shows extremely long MK elongations similar to beads-on-a-thread. Proplatelets and platelets are also visible in the last section of the video.
