# Supplementary material for: Three-Dimensional Environment Sustains Hematopoietic Stem Cell Differentiation into Platelet-Producing Megakaryocytes
Source: PLoS One. 2015 Aug 27;10(8):e0136652. doi: 10.1371/journal.pone.0136652 (PMC4552162; doi:10.1371/journal.pone.0136652)
Supplement: S2 Video — Compared to mature MK obtained from 3D culture, notice the shorter elongations interacting with the surface during the first part of the video and the reduced production of proplatelets and platelets during the second part of the video. Mature and viable MK were recovered from 3D or liquid culture on day 16 and perfused at a shear rate of 1800 s-1 for 45 min on VWF-coated microchannels. Different steps of platelet production were visualized in real-time using the Axiovert 135 transmission optical microscope with 20X Plasdic magnification. Digital images were recorded at 0.25 images/s using Replay software (Microvision Instruments). For visualization, Archimed software (Microvision Instruments) was used to grab frames and record at a velocity of 10 images/sec (40-fold acceleration). Bar = 20 μm. (DOCX) [file pone.0136652.s007.docx]

**Three-dimensional environment sustains hematopoietic stem cell differentiation into platelet-producing megakaryocytes**

Audrey Pietrzyk-Nivau^1^, Sonia Poirault-Chassac^1^, Sophie Gandrille^1,2^, Sidi-Mohammed Derkaoui^3^, Alexandre Kauskot^1^, Didier Letourneur^3^, Catherine Le Visage^3^ and Dominique Baruch^1^

^1^INSERM, UMR-S 1140, University Paris Descartes, Sorbonne Paris Cité, Paris, France

^2^AP-HP, Georges Pompidou European Hospital, Department of Hematology, Paris, France

^3^INSERM, UMR-S 1148, University Paris Diderot, Paris; University Paris Nord, Villetaneuse, Sorbonne Paris Cité, France

ONLINE SUPPLEMENTAL DATA

Short title

Increased 3D megakaryocyte and platelet production

Corresponding author

Dr Dominique Baruch

INSERM UMR-S 1140

4 avenue de l’Observatoire, 75006 Paris, France

Mail: dominique.baruch@parisdescartes.fr

Tel: 33 1 53 73 99 38 / Fax: 33 1 44 07 17 72

Supplemental videos

S1 and S2 Videos

Mature and viable MK were recovered from 3D or liquid culture on day 16 and perfused at a shear rate of 1800 s^-1^ for 45 min on VWF-coated microchannels. Different steps of platelet production were visualized in real-time using the Axiovert 135 transmission optical microscope with 20X Plasdic magnification. Digital images were recorded at 0.25 images/s using Replay software (Microvision Instruments). For visualization, Archimed software (Microvision Instruments) was used to grab frames and record at a velocity of 10 images/sec (40-fold acceleration). Bar = 20 µm.

S2 Video: Platelet production in flow conditions from liquid-culture mature MK

Compared to mature MK obtained from 3D culture, notice the shorter elongations interacting with the surface during the first part of the video and the reduced production of proplatelets and platelets during the second part of the video.
