# Supplementary material for: Three-Dimensional Environment Sustains Hematopoietic Stem Cell Differentiation into Platelet-Producing Megakaryocytes
Source: PLoS One. 2015 Aug 27;10(8):e0136652. doi: 10.1371/journal.pone.0136652 (PMC4552162; doi:10.1371/journal.pone.0136652)
Supplement: S1 Materials and Methods — (DOCX) [file pone.0136652.s008.docx]

**Three-dimensional environment sustains hematopoietic stem cell differentiation into platelet-producing megakaryocytes**

Audrey Pietrzyk-Nivau^1^, Sonia Poirault-Chassac^1^, Sophie Gandrille^1,2^, Sidi-Mohammed Derkaoui^3^, Alexandre Kauskot^1^, Didier Letourneur^3^, Catherine Le Visage^3^ and Dominique Baruch^1^

^1^INSERM, UMR-S 1140, University Paris Descartes, Sorbonne Paris Cité, Paris, France

^2^AP-HP, Georges Pompidou European Hospital, Department of Hematology, Paris, France

^3^INSERM, UMR-S 1148, University Paris Diderot, Paris; University Paris Nord, Villetaneuse, Sorbonne Paris Cité, France

ONLINE SUPPLEMENTAL DATA

Short title

Increased 3D megakaryocyte and platelet production

Corresponding author

Dr Dominique Baruch

INSERM UMR-S 1140

4 avenue de l’Observatoire, 75006 Paris, France

Mail: dominique.baruch@parisdescartes.fr

Tel: 33 1 53 73 99 38 / Fax: 33 1 44 07 17 72

Supplemental materials and methods

Flow cytometry

Expression of the CD34, CD41 and CD42b differentiation markers was assessed between day 6 and day 36 by using a Calibur flow cytometer (BD Biosciences, Le Pont de Claix, France). 3D cells and liquid-culture cells were incubated with fluorescein isothiocyanate (FITC)-conjugated mouse anti-human CD41a (αIIb), R-phycoerythrin (PE)-conjugated mouse anti-human CD42b (GPIbα) (both from Beckman Coulter, Villepinte, France) and allophycocyanin (APC)-conjugated mouse anti-human CD34 (BD Pharmingen, Le Pont de Claix, France) for 30 minutes at 4°C. Controls were prepared with FITC mouse IgG_1_, PE mouse IgG_1_ (both from Beckman Coulter) and APC mouse IgG_1_ (BD Pharmingen). In some experiments, cell viability was assessed with 7-aminoactinomycin D (7-AAD; BD Pharmingen). Results were analyzed with Cell Quest Pro software (BD Biosciences).

Expression of CD11b, CD14 and CD41 differentiation markers was assessed to characterize myelomonocytic populations. 3D cells and liquid-culture cells were incubated with FITC-conjugated mouse anti-human CD14 (BD Pharmingen), PE-conjugated mouse anti-human CD41a and PE-Cy7-conjugated mouse anti-human CD11b (BD Pharmingen). Controls were prepared with FITC mouse IgG_2b_ (BD Pharmingen), PE mouse IgG_1_ and PE-Cy7 mouse IgG_1_ (BD Pharmingen). Expression of CD41 and glycophorin A (GpA) differentiation markers was assessed to characterize erythropoietic populations. 3D cells and liquid-culture cells were incubated with PE-conjugated mouse anti-human CD41a and APC-conjugated mouse anti-human GpA (BD Pharmingen). Controls were prepared with PE mouse IgG_1_ and APC mouse IgG_2b_ (BD Pharmingen). In some experiments, cell viability was assessed with 4',6'-diamidino-2-phenylindole (DAPI, 100 µg/mL; Invitrogen). Acquisitions were made with a BD CANTO 2 flow cytometer and results were analyzed with DIVA software (BD Biosciences).

Megakaryocyte erythroid progenitors (MEP) population was characterized 7 and 23 days after seeding. 3D cells and liquid-culture cells were incubated with FITC-conjugated mouse anti-human CD41a, PE-conjugated mouse anti-human CD45RA, PE-cyanin 7 (PE-Cy7)-conjugated mouse anti-human CD123 (both from BD Pharmingen) and APC-conjugated mouse anti-human CD34 for 30 minutes at 4°C. Controls were prepared with FITC mouse IgG_1_, PE mouse IgG_2b_, PE-Cy7 mouse IgG_2a_ and APC mouse IgG_1_ (BD Pharmingen). In some experiments, cell viability was assessed with DAPI (100 µg/mL). Acquisitions were made with a BD CANTO 2 flow cytometer and results were analyzed with DIVA software.

Ploidy of UCB or peripheral blood CD41^+^/CD42b^+^ cells was studied 11 days after seeding. 3D cells and liquid-culture cells were incubated for 1 hour at 37°C with Hoechst medium (Hoechst 33342, 1 µg/mL; Molecular Probes, Saint-Aubin, France), FITC-conjugated mouse anti-human CD41a and PE-conjugated mouse anti-human CD42b to select a homogenous population of mature MK. Acquisitions were made with a BD LSR II flow cytometer (BD Biosciences). Hoechst staining was analyzed at an emission wavelength of 450 nm and results were analyzed with DIVA software.

Confocal immunofluorescence microscopy

*In situ* cell organization was analyzed 12 days after seeding, without enzymatic treatment. Cells in liquid culture were allowed to attach to L-polylysine coated plates (Thermo-Scientific, Brebières, France) for 20 minutes at 37°C and then fixed with 4% paraformaldehyde (PFA; Alfa Aesar, Karlsruhe, Germany), while cells in 3D were fixed *in situ*. Cells were permeabilized with 0.1% Triton X-100 (Sigma-Aldrich) and treated with RNAse (10 µg/mL; Sigma-Aldrich). Cells were then incubated with YOYO-1 (Cyanin Dimer Y3601, 0.2 µM; Invitrogen) for 1 h at room temperature or with FITC-conjugated mouse anti-human CD41a and PE-conjugated mouse anti-human anti-CD42b overnight at 4°C. Cells were observed with a confocal microscope (Leica System Microscope 510; Zeiss, Gottingen, Germany) equipped with a 40X Plan-NeoFluar objective lens. YOYO-1 and FITC were excited at 491 nm and 488 nm respectively, and their fluorescent emissions were selected with a 505-530 nm bandpass filter. PE was excited at 543 nm and its fluorescence emission was selected with a 560-615 nm bandpass filter. Results were analyzed with LSM Image Browser software (Zeiss).

Clonogenic assay

Colony-forming unit (CFU)-MK potential was assessed using the MegaCult-Collagen kit (Stem Cell Technologies). Sixteen and twenty-three days after seeding, 25 000 cells were plated in wells containing semi-solid MegaCult-C medium. Cells were cultured in the presence of cytokines (50 nM TPO and 20 ng/mL SCF) for 10 days and colonies were quantified as recommended by the manufacturer, using a transmission optical microscope (Leica DM4000 B; Leica Microsystems, Heerbrugg, Germany) equipped with a camera (Leica DFC 420; Leica Microsystems).

RNA extraction and real-time qRT-PCR

Total RNAs were extracted with Trizol (Invitrogen) and extended solvent purification as previously reported [1] and complementary DNAs were synthesized using a commercially available kit (RT core kit; Eurogentec, Angers, France). The expression levels of erythoid Krüppel-like factor (EKLF), c-myb, globin transcription factor 1 (GATA-1), Friend leukemia integration-1 (FLI-1), acute myeloid leukemia-1 (AML-1) and nuclear factor (NF)-E2 were assessed by quantitative polymerase chain reaction (qPCR). All genes were normalized to the housekeeping gene hypoxanthine phosphoribosyltransferase (HPRT). Probes for genes of interest were labeled at the 5’-end with cefamandole (FAM) and those for housekeeping gene with VIC. At the 3’-end, probes were labeled with tetramethylrhodamine (TAMRA; Applied Biosystems, Life Technologies, Saint Aubin, France). Commercial references of the primer mixes used for qPCR are detailed in the S1 Table. PCR amplification was carried out for 10 minutes at 95°C followed by 50 cycles (15 seconds at 95°C and 1 minute at 60°C) in 7900 HT Fast Real Time PCR System (Applied Biosystems). The mRNA expressions were quantified via the ΔΔCt method [2] and expressed in percentage compared with control.

Platelet production in flow assay

Cell suspensions were flown in microchannels by means of a pressure controller integrated in a BioFlux200 microfluidic platform (Fluxion Biosciences, South San Francisco, CA) [3, 4]. Microchannels were rinsed with 150 mM NaCl, 50 mM Tris-HCl buffer (pH 7.4) perfused at a shear stress of 20 dyn/cm^2^ for 15 s, and then coated with human VWF (40 µg/mL; Wilfactin, Laboratoire Français du Fractionnement et des Biotechnologies, Lille, France) by perfusion at 20 dyn/cm^2^ for 15 s and incubation in the absence of flow overnight at 4°C, before a final rinse with PBS for 10 min at 20 dyn/cm^2^. Mature and viable MK in complete medium with a viscosity of 1 cP (0.98-1.05 cP) were perfused at 18 dyn/cm^2^ for 45 min in the microchannel (S1 Fig.). The chamber was then rinsed with PBS containing 1.25 mM EDTA for 30 min at 18 dyn/cm^2^. Real-time platelet production was recorded at 20X Plasdic magnification using a transmission optical microscope (Axiovert 135; Zeiss, Le Pecq, France) connected with a CCD camera (Q-click Imaging). Proplatelets and platelets were counted in the microchannel by using Histolab software (Microvision Instruments, Evry, France) [5].

Platelet activation and spreading

Platelet-forming MK and platelet suspensions were collected at the outlet of the flow chamber and centrifuged for 10 min at 1240 *g* (S1 Fig.). The pellet was deposited on BSA density gradient and centrifuged for 15 min at 80 *g* to separate mature MK from platelets [6]. Fractions containing platelets were centrifuged for 10 min at 1240 *g*. Platelets were suspended in Hepes-Tyrode buffer [7] containing 2 mM CaCl_2_. On the one hand, platelet suspensions were either not activated or activated with thrombin (1 U/mL; Sigma-Aldrich) for 10 minutes of preincubation. Platelets were then added to fibrinogen (200 µg/mL; Hyphen BioMed, Neuville-sur-Oise, France)-coated glass ibidi chamber slides (Biovalley, Marne-La-Vallée, France). CD41a/phalloidin staining was then performed as previously described [5]. The coverslips were mounted in DABCO mounting solution (Dako, Trappes, France). CD41/F-actin staining was observed at 63X Plasdic magnification using a fluorescence optical microscope (Axio Observer D1; Zeiss; S1 Fig.). On the other hand, platelet suspensions were either not activated or activated with PAR1-activated peptide (TRAP-6, 10 µM; Bachem, Bubendorf, Switzerland) and directly added to fibrinogen (20 µg/mL)-coated glass ibidi chamber slides. Platelets were then incubated with FITC-conjugated mouse anti-human PAC-1 (BD Biosciences) for 10 minutes to stain the activated integrin αIIbβ3, fixed with 4% PFA, permeabilized with 0.1% Triton-X100 and incubated with AlexaFluor 488-conjugated goat anti-mouse IgM (BD Biosciences) for 45 minutes [8, 9]. Finally, platelets were incubated with AlexaFluor 546-phalloidin (Invitrogen) for 20 minutes to stain F-actin. The coverslips were mounted in DABCO mouting solution and activated integrin αIIbβ3/F-actin staining was observed at 63X Plasdic magnification with Axio Observer D1 (S1 Fig.).

Statistic data analysis

Results are expressed as means of independent experiments ± standard error of the mean (SEM). Statistical significance was evaluated with Mann-Withney test, using StatView statistical software. p values <0.05 were considered significant.

References

1. Yu C, Young S, Russo V, Amsden BG, Flynn LE. (2013) Techniques for the isolation of high-quality RNA from cells encapsulated in chitosan hydrogels. Tissue Eng Part C Methods 19(11): 829-838.

2. Livak KJ, Schmittgen TD. (2001) Analysis of relative gene expression data using real-time quantitative PCR and the 2(-Delta Delta C(T)). Method Methods 25(4): 402-408.

3. Conant CG, Schwartz MA, Nevill T, Ionescu-Zanetti C. (2009) Platelet adhesion and aggregation under flow using microfluidic flow cells. J Vis Exp (32): 1644

4. Conant CG, Schwartz MA, Beecher JE, Rudoff RC, Ionescu-Zanetti C, Nevill JT. (2011) Well plate microfluidic system for investigation of dynamic platelet behavior under variable shear loads. Biotechnol Bioeng 108(12): 2978-2987.

5. Dunois-Larde C, Capron C, Fichelson S, Bauer T, Cramer-Borde E, Baruch D. (2009) Exposure of human megakaryocytes to high shear rates accelerates platelet production. Blood 114(9): 1875-1883.

6. Robert A, Cortin V, Garnier A, Pineault N. (2012) Megakaryocyte and platelet production from human cord blood stem cells. Methods Mol Biol 788: 219-247.

7. Poirault-Chassac S, Nguyen KA, Pietrzyk A, Casari C, Veyradier A, Denis CV, et al. (2013) Terminal Platelet Production is Regulated by Von Willebrand Factor. PLoS One 8(5): e63810.

8. Mazharian A, Roger S, Berrou E, Adam F, Kauskot A, Nurden P, et al. (2007) Protease-activating receptor-4 induces full platelet spreading on a fibrinogen matrix: involvement of ERK2 and p38 and Ca2+ mobilization. J Biol Chem 282(8): 5478-5487.

9. Jirouskova M, Jaiswal JK, Coller BS. (2007) Ligand density dramatically affects integrin alpha IIb beta 3-mediated platelet signaling and spreading. Blood 109(12): 5260-5269.
